# Protocol for recording visual maps in the mouse superior colliculus and visual cortex with intrinsic optical imaging

**DOI:** 10.1016/j.xpro.2026.104430

**Published:** 2026-03-12

**Authors:** Flora Boutet-Porretta, Josien Visser, Arthur Matthys, Armelle Rancillac, Nathalie Rouach, Jérôme Ribot

**Affiliations:** 1 Center for Interdisciplinary Research in Biology, Collège de France, CNRS, INSERM, PSL-Neuro, Université PSL, Paris, France; 2 Doctoral School N°158, Sorbonne Université, Paris, France

**Keywords:** Microscopy, Cognitive Neuroscience, Systems biology

## Abstract

Intrinsic optical imaging (IOI) is a powerful approach to record visual-functional maps across the mouse superior colliculus (SC) and primary visual cortex (V1). Here, we present a protocol to map retinotopy and orientation preference in the SC within 15 min using periodic stimulation and Fourier analysis. We also describe how to obtain ocular dominance maps in V1 from independent monocular stimulation. We provide detailed instructions for surgical preparation, imaging setup, and preprocessing techniques.

For complete details on the use and execution of this protocol, please refer to Visser et al.^1^

## Highlights

• Step-by-step instructions for SC surgical access and through-skull imaging of V1
• Implementing, performing, and analyzing intrinsic optical imaging data
• Using periodic visual stimulation to compute retinotopic phase maps

## Abstract (editor highlights)

Publisher’s note: Undertaking any experimental protocol requires adherence to local institutional guidelines for laboratory safety and ethics.

## Graphical abstract

**Figure:**
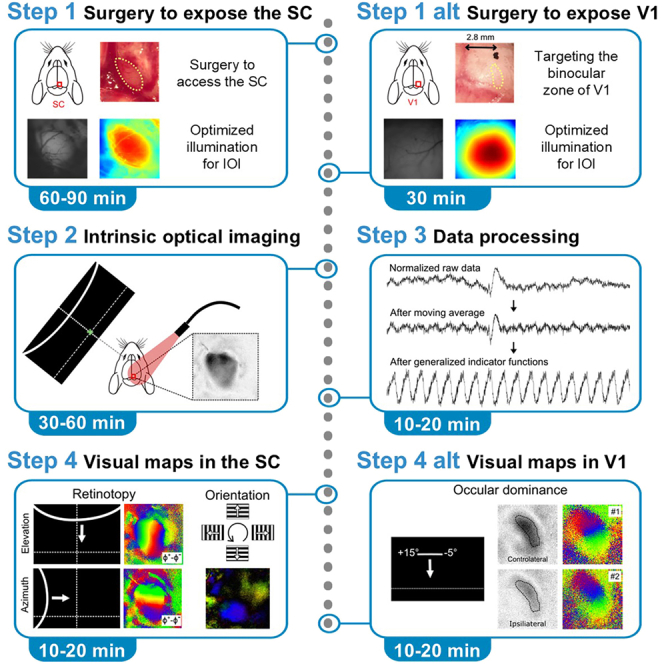

## Before you begin

Intrinsic optical imaging (IOI) is a non-invasive technique widely used to map brain functional architecture by monitoring light reflectance changes linked to neural activity. These signals arise from local variations in blood volume, oxygenation, and light scattering.^2^^,^^3^ Because it does not rely on exogenous indicators or dyes, IOI enables long-term recordings without altering the underlying cellular physiology. It is particularly suited for imaging large cortical regions that can span several millimeters.^1^^,^^4^^,^^5^ While initially developed in cats and non-human primates,^6^^,^^7^ IOI has since been extensively used in rodents to study sensory processing, cortical topography, and experience-dependent plasticity.^5^^,^^8^^,^^9^^,^^10^^,^^11^^,^^12^^,^^13^^,^^14^^,^^15^^,^^16^^,^^17^

The present protocol provides a detailed procedure for using periodic IOI and Fourier analysis to map retinotopy, orientation preference, and ocular dominance in the mouse visual system. It includes optimized steps for surgical preparation to ensure optical access to either the superior colliculus (SC) or the primary visual cortex (V1), as well as stimulus presentation, image acquisition and pre-filtering methods. This protocol is suitable for studying visual map organization and plasticity in wild-type or genetically modified mice, and is designed to ensure reproducibility across sessions, animals, and experimental conditions.

### Innovation

Functional maps obtained with IOI have traditionally relied on episodic stimulation, where each stimulus condition is presented in discrete trials and responses are averaged across repetitions, requiring one to several hours of acquisition time.^4^^,^^18^ In contrast, recent developments based on periodic stimulation have drastically reduced the acquisition time to just a few minutes.^5^^,^^13^^,^^14^^,^^19^ In this paradigm, a continuously cycling stimulus is presented to the animal, and intrinsic signals are analyzed in the frequency domain using Fourier transform. Because acquisition is faster, the approach is more robust to slow drifts in the recording signals. Moreover, as only the signal component at the stimulus frequency is extracted, much of the physiological noise is inherently rejected. Additional pre-filtering methods can further enhance signal-to-noise ratio and map quality.^5^^,^^20^^,^^21^^,^^22^ This approach therefore represents a practical and robust advancement over traditional episodic IOI paradigms.

### Institutional permissions

All experiments were performed in accordance with the European Communities Council Directives of 01/01/2013 (2010/63/EU) for animal care and experimentation and of the French ethic committee (ethics approval #201902121059308 and #2022112516059564 delivered by the French Ministry of Higher Education, Research and Innovation).

### Preparation of the equipment for intrinsic optical imaging

**Timing: 20 min**

1. Prepare the imaging setup for intrinsic optical signal acquisition (Figure 1A):
  a. CCD camera (e.g., Dalsa 1M60) equipped with a tandem lens system (e.g., 135 × 50 mm, Nikon) for wide-field imaging.
  b. White LED light source (e.g., VCM-D1, Vincent Associates) connected to a flexible light guide and power control system (e.g., Oriel 69931, Newport) to adjust light intensity during surface imaging and intrinsic signal acquisition.
    ***Note:*** Optical filters of different wavelengths can be inserted to adjust illumination depending on the imaging phase. For instance, green light facilitates the visualization of superficial blood vessels, while longer wavelengths, typically in the red to near-infrared range, are used to record intrinsic optical signals.
  c. Stereotaxic frame with ear and mouth bars to ensure stable head fixation throughout the imaging session.
  d. Thermoregulated heating pad with a rectal probe to maintain stable body temperature between 35°C–37°C during anesthesia.

**Figure 1:**
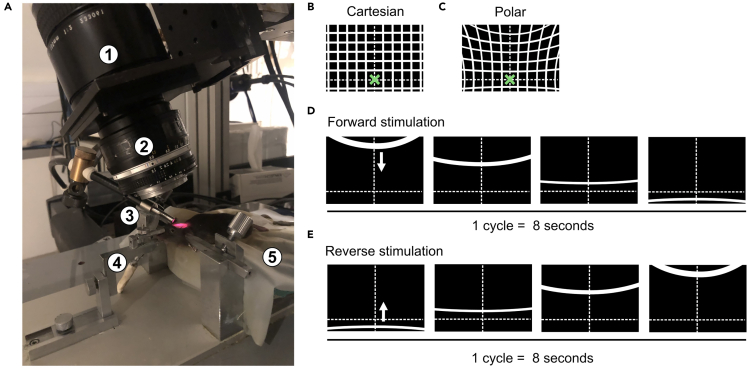
Intrinsic optical imaging setup and retinotopic stimulation paradigm (A) Imaging setup for intrinsic optical signal acquisition in head-fixed mice. The setup includes (1) and (2) a 135 × 50 mm tandem lens system (Nikon), (3) a high-power light source directed at the exposed brain, (4) a stereotaxic frame with mouth bar and ear bars for stable head fixation, (5) a temperature-controlled heating pad, and a Dalsa 1M60 CCD camera mounted on the lens assembly (not visible on the picture). (B) Cartesian coordinate representation of the visual stimulation monitor. The spatial layout of visual stimuli is shown in original screen-based coordinates. The green cross indicates the minimal distance between the mouse eye and the screen (20 cm). (C) Polar coordinate transformation of the stimulus monitor. The originally Cartesian stimulus layout is converted into polar visual space centered on the contralateral eye. The curved, quasi-vertical and horizontal lines represent visual eccentricities (i.e., iso-eccentricity contours) projected from spherical visual space onto the flat monitor surface. (D) Example of a forward stimulation cycle. The stimulus begins at the highest vertical eccentricity (top of the screen in polar space) and progressively shifts downward toward the lowest eccentricity at the bottom. Each cycle lasts 8 seconds, and the stimulus sweeps smoothly through the full visual range along the vertical meridian. See also Methods video S1. (E) Same periodic stimulation as in (D), but presented in the reverse direction, starting at the lowest eccentricity and sweeping upward to the highest.
2. Prepare the visual stimulation and data acquisition system:
  a. Stimulation monitor (e.g., LCD, 21″, 60 Hz, DELL).
  b. Image acquisition and synchronization system capable of acquiring high-speed data (10 Hz) and synchronizing data acquisition with visual stimulus presentation.

**CRITICAL:** An acquisition rate of 10 Hz is not inherently high and is typically suitable only for cameras with a high electron well depth. If the camera has a low well depth, a substantially higher acquisition rate should be used, combined with the longest possible exposure time, in order to ensure a sufficient signal-to-noise ratio for intrinsic signal imaging. See, for example, Augustinaite and Kuhn^12^ for a detailed ISI protocol.

**CRITICAL:** The stimulation and imaging software are often on two different computers. In this case, they need to be synchronized to ensure temporal consistency of the analysis afterwards. Make sure that when the recording starts, it triggers the stimulation on the screen.

3. Generate the programs for the visual stimulation programs (e.g., CRS toolbox for ViSaGe within MATLAB).

***Note:*** For the study of retinotopy, the stimulation consists of a white bar (2° width) drifting along the vertical (elevation) or horizontal (azimuth) axis of the screen. The stimulus sequence lasts 8 s and is presented periodically 30 times during the whole session, following the periodic stimulation paradigm introduced by Kalatsky and Stryker.^5^

***Note:*** For orientation preference mapping, drifting sinusoidal gratings are presented while their orientation is rotated continuously and periodically over 30 s, either clockwise or counterclockwise, as described previously.^5^ The drift rate of the gratings is set to 1.5 Hz and the spatial frequency to 0.015 cycles per degree.

***Note:*** For ocular dominance mapping, a white bar (2° width) drifting periodically along the vertical axis is presented independently to each eye while the other eye is occluded. The stimulus is restricted to the binocular visual field (from −5° in the ipsilateral field to +15° in the contralateral field) and is presented in alternating blocks of contralateral and ipsilateral eye stimulation, following established protocols for ocular dominance mapping in mice.^8^^,^^14^

***Note:*** Because of the short distance between the eye and the screen, the stimulus may appear distorted (e.g., compressed at the periphery). To ensure spatial uniformity across the visual field, the screen’s Cartesian coordinates can be converted into polar visual space, with eccentricities projected appropriately onto the flat display (Figures 1B and 1C). This transformation preserves stimulus dimensions defined in degrees of visual angle across the visual field. For example, it maintains a constant bar width of 2° as used for retinotopic mapping, thereby avoiding eccentricity-dependent distortions inherent to Cartesian coordinates. In these new coordinates, the stimulus for mapping elevation is shown for both forward and reverse motion (Figures 1D and 1E and Methods video S1).

Methods video S1. Time-resolved hemodynamic response to visual stimulation, related to step 17 **Left:** Visual stimulus consisting of a white bar sweeping downward across the visual field in polar coordinates over 8 seconds. **Right**: Intrinsic optical signal recorded from the superior colliculus (SC) in response to the stimulus. Each frame corresponds to one time point in the periodic stimulation cycle (80 frames total; 10 frames per second).

### Preparation of surgical tools and reagents for cranial surgery

**Timing: 30 min**

4. Prepare the surgery tools.
  a. Sterilize all surgical instruments, consisting of scissors, forceps and tweezers, using an autoclave or a bead sterilizer.
  b. Curve the tip of a 26-gauge needle using tweezers to form a fine hook. This will be used to gently remove the dura mater after the craniotomy.
  c. Cut the tip of a 20-gauge needle and attach it to a 1 mL syringe. Cut the distal end of the syringe barrel and insert it into silicone aspiration tubing (inner diameter ∼5 mm, outer diameter ∼8 mm) connected to a vacuum pump to enable fine cortical aspiration during surgery.

***Note:*** After cutting, the tip of the 20-gauge needle may become flattened, narrowing the aperture and limiting suction. Gently press the tip at various angles using scissors to reshape and round off the aperture.

***Alternatives:*** Commercially available tools may be used if preferred.

5. Sterilize the surgical environment. Wipe down the stereotaxic frame, surgical platform, and tool table with 70% ethanol.
6. Turn on the heating pad. Ensure the pad has sufficient time to reach the target temperature of 37°C before placing the animal.

***Note:*** Place the rectal temperature probe under the pad so it reflects the surface warming properly. If left exposed to air, the pad may overheat.

7. Prepare required solutions.
  a. Dilute anesthetics and supporting drugs: urethane in sterile 0.9% NaCl (100mg/mL), chlorprothixene in sterile water (4mg/mL) and atropine in sterile water (0.05mg/mL).
  b. Prepare a 2.5% agarose solution in 0.9% NaCl solution. Heat the solution gently (e.g., using a microwave or water bath) until the agarose is fully dissolved and clear.
    ***Note:*** You can prepare large volumes of agarose solution in advance, aliquot if necessary, and store at 4°C for reuse. Reheat gently before each experiment to avoid degradation.
  c. Pour cold Ringer’s solution (Ringer lactate) into a beaker to have it readily available during surgery. Do the same with 0.9% NaCl solution.
    ***Note:*** Store the stock Ringer's solution at 4°C and fill the beaker with it when needed.
8. Perform a pre-surgical checklist. Visualize the entire procedure step-by-step and confirm that all required drugs, tools and consumables are readily available and within reach.

## Key resources table

**Table:** 

REAGENT or RESOURCE	SOURCE	IDENTIFIER
**Chemicals, peptides, and recombinant proteins**		

Urethane	Sigma-Aldrich	Cat# U2500
Chlorprothixene hydrochloride	Sigma-Aldrich	Cat#1671
Agarose	Fisher Scientific	BP1360-100
Ringer Lactate	Osalia	https://med-vet.fr/produits/medicament/ringer-lactate-osalia-solution-pour-perfusion/38d3e44f-3c29-4313-99d8-a86f5815492e
Atropine	Sigma-Aldrich	Cat# Y0000878
Dexamethasone	MSD	https://www.msd-animal-health-hub.co.uk/Products/Dexadreson
NaCl solution (0.9%)	CDM Lavoisier	Cat# 201178

**Deposited data**		

Raw and analyzed data	This paper	https://github.com/tchetch12/IOI_Retinotopy_Analysis

**Experimental models: Organisms/strains**		

Mouse C57BL/6J (aged 1-2 months, both males and females)	Charles River	RRID: IMSR_JAX:000664

**Software and algorithms**		

Longdaq acquisition software	Optical Imaging, Inc.	–
CRS toolbox for ViSaGe	Cambridge Research Systems	https://www.crsltd.com/tools-for-vision-science/visual-stimulation/visage/
MATLAB (Version r2023b)	MathWorks	RRID:SCR_001622

**Other**		

Microtorque II Micro Drill	Ram Products	–
Light Power Suply	Newport	https://www.artisantg.com/Scientific/67214-1/Newport-Oriel-69931-Digital-Radiometric-Power-Supply
CCD camera	Dalsa	1M60
Light shutter driver and controller	Vincent Associates	VCM-D1
Cover slips	Epredia	12 mm diameter
Hemostatic sponge (Spongel)	Ethicon	Spongostan
Aquagel	Parker Laboratories	TB-142-0000G
Surgical tools	Fine Science Tools	Standard microsurgery kit
Homeothermic monitoring system	Harvard Apparatus	50-7220-F

## Step-by-step method details

### Preparation of the mouse for cranial surgery

**Timing: 30 min**

**Figure 2:**
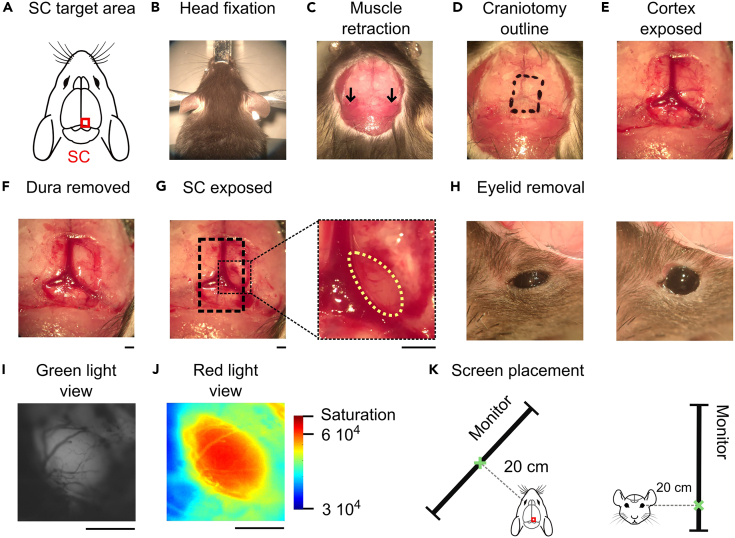
Surgical procedure, illumination and screen position for intrinsic optical imaging of the superior colliculus (A) Schematic representation of the superior colliculus (SC) and its exposure for imaging. (B) Mouse positioned in a stereotaxic frame using ear bars and a mouth bar for head fixation. (C) Zoomed view of the skull following scalp incision. Black arrows indicate the position of the neck muscles to be dissected and retracted to clear access to the posterior skull. (D) Delineation of the craniotomy window (∼5 × 8 mm), drawn over the skull above the anterior SC, just right of the lambda. (E) Photograph of the cortical surface and blood vessels following the craniotomy. (F) Same view as (E) after removal of the dura mater, revealing the cortical surface. (G) Exposure of the SC after cortical aspiration. The right panel shows a zoomed-in view highlighting superficial vasculature on the SC surface. (H) View of the contralateral eye before (left) and after (right) excision of the upper and lower eyelids to ensure an unobstructed visual field during stimulation. (I) Image of the SC acquired under green light illumination (vascular contrast mode) using the imaging system. (J) Same view under red light illumination optimized for intrinsic signal acquisition. (K) Illustrations showing the positioning of the visual stimulation screen relative to the animal’s head and contralateral eye. Scalebar: 1 mm.
4. Insert the rectal temperature probe.
  a. Dip the tip of the probe (∼1 mm diameter) into Aquagel lubricant gel.
  b. Insert the probe.
  c. Cover the animal with a paper towel to reduce heat loss.

***Note:*** Maintain body temperature at 37°C throughout the whole procedure.

***Note:*** Secure the probe (e.g. with tape) to minimize displacement during surgery.

1. Weigh the animal and calculate appropriate anesthetic dosages.
2. Inject the animal and monitor for anesthesia induction.
  a. Inject chlorprothixene (8 mg/kg, intramuscular) and urethane (1.2 g/kg, intraperitoneal) to induce anesthesia.
    ***Note:*** Due to its carcinogenic and mutagenic properties, urethane must be used exclusively for terminal procedures.
    ***Alternatives:*** To perform IOI as part survival surgery, alternative anesthetics can be used, such as isoflurane^23^ or injectable anesthetic mixtures (e.g., Medetomidine/Midazolam/Butorphanol^12^).
  b. Inject atropine (0.1 mg/kg) and dexamethasone (2 mg/kg) subcutaneously to reduce bronchial secretions and prevent vagal reflexes (atropine), and to minimize inflammation and cerebral edema (dexamethasone).
  c. Once the animal stops moving, transfer it to the heating pad to prevent hypothermia.
  d. Monitor for progressive slowing of the respiratory rate and assess loss of reflexes via toe pinch to confirm adequate depth of anesthesia before proceeding.
    ***Note:*** Full anesthesia typically develops within 20–30 min.
    ***Note:*** The depth of anesthesia should be checked regularly throughout the procedure.
3. Position the upper front teeth in the opening of the tooth bar. Head-fix the mouse in the stereotaxic frame. Gently secure the head using ear bars and the nose clamp (Figure 2B).

5. To prevent dehydration of the cornea, apply a drop of 0.9% NaCl onto each eye using a syringe.

**CRITICAL:** Continuously check the eyes during the surgery and reapply 0.9% NaCl as needed. Corneal dehydration is indicated by whitening or opacity, which may compromise visual stimulation efficacy in later steps. Troubleshooting problem 1.

***Alternatives:*** Commercially available ophthalmic ointments or mineral oils can be used.

### Surgical procedure to expose the superior colliculus

**Timing: 60**–**90 min**

This procedure details the steps for exposing the SC prior to imaging (Figure 2A).

6. Remove the scalp skin (Figure 2C).
  a. Gently pinch the skin above the skull using tweezers and make an incision using scissors of approximately 1 cm.
  b. Thoroughly remove any connective tissue and remaining hair on the skull using a combination of salt solution and cotton swabs while pushing the excessive skin aside to expose the full skull surface. Clean and dry the exposed area.
  c. Carefully incise the neck muscle on each lateral insertion with small scissors and push it away to free the posterior area of the bone.

**CRITICAL:** Considering the posterior location of the SC, exposing a large bone is necessary to avoid soft tissue entrapment in the drill during the craniotomy and to minimize the risk of tearing.

***Note:*** Bleeding after muscle incision is common and can be stopped by applying pressure with a sterile tissue (Kimtech) for several seconds.

7. Define the position of the craniotomy for the right SC imaging (Figure 2D).
  a. Identify the SC, located just right of lambda, near the intersection of cranial sutures.
  b. Using lambda as a reference point, draw a ∼5 × 8 mm rectangle over the skull, centered approximately 0.5 mm lateral and 0.5–1 mm anterior to lambda. This window should encompass the anterior portion of the right SC.

**CRITICAL:** The craniotomy must be large enough to provide ample anterior and lateral access for cortex aspiration and avoid accidental damage to the sagittal or transverse sinus during drilling, which can result in fatal hemorrhage.

***Note:*** To image the left SC, mirror the craniotomy across the midline by shifting the window to the left hemisphere. Using lambda as a reference, position the craniotomy approximately 0.5 mm lateral (left) and 0.5–1 mm anterior to lambda, preserving the same dimensions (∼5 × 8 mm).

8. Using a 1 mm drill bit, drill along the outlined rectangle until the bone flexes upon gently pressure with forceps onto the inner rectangle. This indicates that the bone is sufficiently weakened. Stop before reaching the underlying soft tissue to avoid damaging the brain.

**CRITICAL:** Move the drill head slowly along the outline to avoid concentrating force or heat on a single point.

**CRITICAL:** To prevent heat buildup, interrupt drilling every few seconds to apply cold saline solution. Allow it to cool briefly, then gently dry the surface before resuming.

**CRITICAL:** Avoid drilling over visible blood vessels within the skull.

**CRITICAL:** The craniotomy should always be covered with saline solution to prevent dehydration of the brain.

***Note:*** Minor bleeding from skull vessels may occur. Apply cold Ringer solution using a 1 mL syringe and press gently with Kimtech to stop the bleeding.

***Note:*** Use the tips of tweezers to gently press on the bone edges, assess thinning and decide whether to continue or stop drilling. Tweezers can also be used to gently fracture the bone once it is sufficiently thinned.

9. Remove the bone flap (Figure 2E).
  a. Apply cold Ringer solution and let it rest for about 1 minute.
  b. Slide a fine-tipped tweezers beneath one corner of the bone to be removed.
  c. Gently lift the bone flap upward to detach it from the dura mater.

**CRITICAL:** Resistance when lifting the flap indicates that bone edges remain too thick. Do not force. Instead, return with the drill and thin further.

**CRITICAL:** Sudden detachment of the bone flap could rupture large blood vessels, leading to profuse bleeding. In this case, use repeated application of cold ringer solution and removal of the fluid using Kimtech tissue. If bleeding persists, place a Spongel pad moistened with Ringer’s solution directly over the site, then cover it with Kimtech to maintain pressure and absorb blood. Troubleshooting problem 2.

10. Remove the dura mater (Figure 2F).
  a. Use the curve-tip needle to gently incise the dura near the edges of the transverse and sagittal sinuses.
  b. Push the dura away to expose the cortical surface.

***Alternatives:*** The incision can be made more anteriorly, and the dura can be gently retracted posteriorly toward the sinuses.

***Note:*** Fine tweezers may assist in lifting the dura.

11. Aspirate the overlying cortex to expose the SC (Figure 2G). Use the aspiration system (20G needle connected to vacuum pump) to carefully remove the cortex overlaying the anterior SC.

***Note:*** Gently pushing the cortex anteriorly, away from the transverse sinus, can help detach it more easily and reduce tension during aspiration.

**CRITICAL:** The visual cortex lies above the SC, but the two structures are separated by a thin anatomical plane. During aspiration, gently pull the cortex upward to visually identify the boundary before proceeding. Perform aspiration progressively to avoid penetrating too deeply. Damage to the superficial layers of the SC can indeed impair subsequent imaging.

***Note:*** The surface of the SC is recognizable by the presence of superficial small blood vessels. Troubleshooting problem 3.

***Note:*** The most anterior part of the SC lies deeper in the brain than the posterior part. Adapt hand motion and depth accordingly during aspiration.

12. Apply agarose and seal the imaging chamber.
  a. Heat the 2.5% agarose solution (e.g., in the microwave) until fully liquefied, then allow it to cool to ∼37–40°C before applying onto the brain tissue.
  b. Apply a drop of warm agarose onto the exposed surface of the SC using a 1mL syringe.
    **CRITICAL:** Before applying to the brain, test a small drop of agarose solution on the back of your hand to ensure that it is not too hot. Excessive heat can damage the tissue and cause bleeding.
  c. Place a 1cm diameter glass coverslip onto the agar with tweezers, centering it over the SC.
  d. Apply additional agarose around the edges of the coverslip to seal and stabilize the glass window.
13. Remove the eyelid of the contralateral eye (Figure 2H). Using fine scissors and tweezers**,** gently excise the upper and lower eyelid of the eye contralateral to the imaged hemisphere to ensure an unobstructed visual field during stimulation.

***Note:*** Minor bleeding may occur. If so, apply gentle pressure with a Kimtech tissue until the bleeding stops.

### Preparation of the imaging setup

**Timing: 25 min**

This procedure details the steps required to configure the imaging system and visual stimulation screen before intrinsic signal acquisition (Figures 2I–2K).

14. Focus the camera on the surface of the SC and define a region of interest (ROI).
  a. Launch the image acquisition software used for data collection.
  b. Start live imaging to visualize the field of view using the grayscale display.
    ***Note:*** The green filter is used to visualize the superficial vasculature to locate the surface of the SC.
  c. Position the lamp above the head, oriented toward the SC to evenly illuminate the area of interest without blocking the camera view.
  d. Increase the camera gain to enhance vascular contrast and improve visibility.
  e. Adjust the camera’s position in X, Y and Z until surface blood vessels over the SC are clearly visible and in focus (Figure 2I).
  f. Open the ROI selection tool within the acquisition software.
  g. Draw a rectangle around the SC and confirm the selection.
    ***Note:*** The SC should be centered within the ROI. Use a sufficiently large rectangle to cover the full surface area of the SC.
    ***Note:*** An image of the selected ROI can be saved for later verification or visualization of the chosen region.
15. Define optimal illumination for the recording session (Figure 2J).
  a. Lower the camera focus by approximately 250 μm for intrinsic signal acquisition.
  b. Replace the green filter with the red filter (700 nm).
    ***Alternatives:*** While 700 nm is commonly used, other wavelengths within the 610–700 nm range can also be used for intrinsic signal imaging, depending on the desired trade-off between tissue penetration and sensitivity to hemodynamic changes.
  c. Reset the camera gain to baseline and select a display mode or lookup table that allows visualization of signal dynamic range and saturation.
    ***Note:*** Visualization of signal intensity and saturation levels facilitates precise adjustment of illumination to avoid saturation and ensure homogeneous illumination across the ROI.
  d. Adjust the lamp position so that the ROI is illuminated as uniformly as possible.
  e. Use the lamp controller to set the desired light intensity.
    **CRITICAL:** Avoid reaching full saturation. Keep a safety margin of ∼5–10% below the saturation threshold, as slow baseline fluctuations during acquisition can cause overexposure and signal clipping.
    ***Note:*** Two light sources can be used to ensure even illumination across the whole region.
  f. Once the illumination is optimized, lock the acquisition and illumination settings.
  g. Shield the ipsilateral eye to ensure that only the contralateral eye receives visual stimulation.
    ***Note:*** The shield can be made from a piece of black cardboard attached to a small support, which is placed in front of the eye to be covered so that the cardboard blocks vision in that eye. Commercially available alternatives can also be used.
16. Position the visual stimulation screen and acquisition (Figure 2K).
  a. Place the LCD stimulation monitor approximately 20 cm from the contralateral eye, with an angle of around 45 degrees with the vertical meridian (Figure 2K, left panel).
    ***Note:*** In our setup, we used a 21″ monitor covering ∼110° of azimuth and ∼60° of elevation in visual space.
  b. Ensure that the screen elevation matches the coordinates used during visual stimulation design, especially when working in polar coordinates (Figure 2K, right panel; see Figures 1B and 1C for projection geometry).
  c. Once the screen is positioned, wait approximately 5 minutes to allow the animal to adapt to complete darkness before starting the stimulation and acquisition.
    ***Note:*** Once the screen is correctly positioned, consider marking a reference point on the stereotaxic frame. This helps ensure consistent placement in future experiments and enhances reproducibility.
  d. Start data acquisition for retinotopic mapping. Depending on the condition, the stimulus may sweep along the elevation (as shown in Figures 1D and 1E) or azimuth axis. Each stimulation direction (forward and reverse) is presented in succession and lasts 4 minutes, corresponding to 30 full cycles presentation. Images are acquired at 10 Hz with a spatial resolution of approximately 10.5 μm per pixel.

### Analysis of the retinotopic map from forward and reverse stimulations

**Timing: 5**–**10 min**

This section describes how to process the intrinsic optical signals acquired during periodic stimulation to compute retinotopic phase maps and apply appropriate pre-filtering methods. As an example, we detail the processing of elevation maps, based on visual stimuli presented in Figures 1D and 1E.

**Figure 3:**
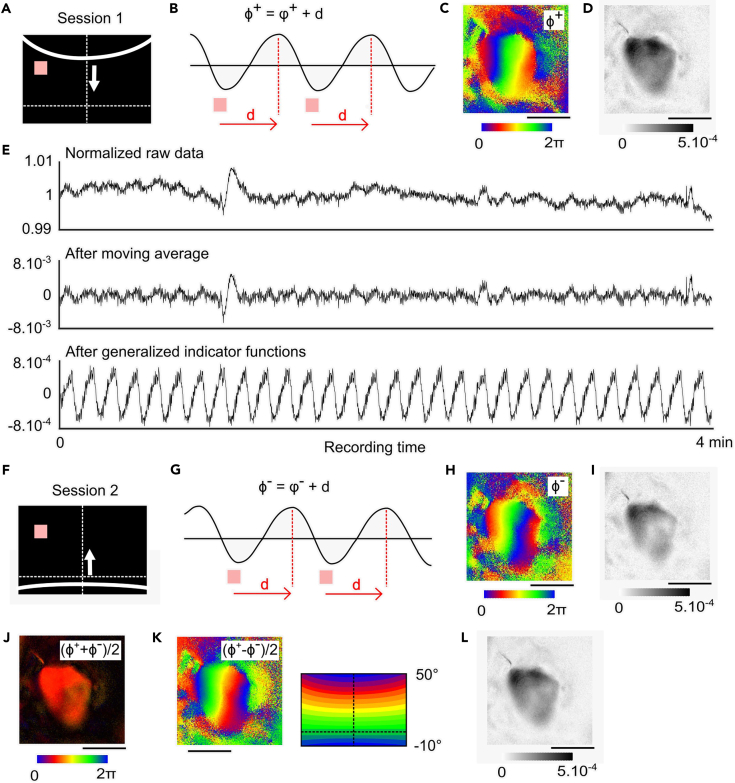
Fourier-based mapping of retinotopy in the superior colliculus (A) Visual stimulation screen during the forward stimulation paradigm. The red square indicates the receptive field (RF) location of a representative neuron in the superior colliculus (SC). (B) Schematic response evoked as the optimal periodic stimulus crosses the receptive field shown in (A). The intrinsic signal exhibits a periodic profile, reaching its maximum after a hemodynamic delay d. The resulting measured phase Φ^+^ at peak amplitude reflects the sum of the true neuronal response phase ϕ^+^ and the delay, such that Φ^+^ = ϕ^+^ + d. (C) Resulting phase map Φ^+^ computed from the Fourier transform of the pixelwise signal during forward stimulation, showing retinotopic organization (plus hemodynamic delay) based on stimulus timing. (D) Corresponding magnitude map, indicating the strength of the periodic response at the stimulus frequency for each pixel. (E) Comparison of various pre-filtering methods applied to the temporal signal at a single pixel. Top: Raw signal normalized by the temporal mean. Middle: Signal after moving average filtering. Bottom: Signal after processing with the generalized indicator function method. (F–I) Same as (A–D), but for the reverse stimulation paradigm, yielding the measured phase map Φ^-^ and its associated magnitude. (J) Delay map computed as (Φ^+^ + Φ^-^)/2, which reflects the hemodynamic delay across the visual field. (K) Retinotopic phase map computed as (Φ^+^ − Φ^-^)/2, which is free of the hemodynamic delay. (L) Magnitude map corresponding to the absolute phase difference, showing the strength of the corrected signal. Scalebar: 1 mm.
18. Map Reconstruction from Reverse Stimulation (Figures 3F–3I).
  a. Repeat the exact processing steps described in Step 17, this time using the dataset from the reverse stimulation (Figures 3F and 3G).
  b. This yields the phase map ϕ^-^ = φ^-^ + d, where φ^-^ is the phase corresponding to the reverse sweep, and the associated magnitude map (Figures 3H and 3I).

***Note:*** Because the stimulus direction is reversed, φ^+^= - φ^-^.

17. Map Reconstruction from Forward Stimulation (Figures 3A–3E).
  a. Load the raw intrinsic optical signal recording sequence corresponding to the full stimulation duration.
  b. Normalize each pixel time series by dividing by its temporal mean (Figure 3E, top panel).
  c. Apply a filtering method.
    **CRITICAL:** Pre-filtering is essential to reduce noise and preserve the integrity of the phase map. Figures 3E and S1 compare different strategies, such as moving average filtering (middle panel) proposed by Kalatsky & Stryker^5^ or the Generalized Indicator Function (GIF, bottom panel) filtering.^20^ The latter method isolates frequency components more selectively (Figure 3E), preserving signal integrity across time segments and improving reproducibility of retinotopic maps (Figure S1).
    ***Note:*** To visualize the dynamics of the hemodynamic response following this pre-filtering step, Methods video S1 shows the time-resolved activity evoked by the visual stimulus displayed concurrently on the left.
  d. Perform Fast Fourier Transform (FFT) on the pixelwise signal and extract the complex component at the stimulus frequency (e.g., 0.125 Hz for an 8 s period).
    ***Note:*** The complex component extracted from Fourier analysis contains two key pieces of information: the amplitude, which reflects the strength of the response at the stimulus frequency, and the phase ϕ^+^, which reflects the time of the hemodynamic response peak. This phase ϕ^+^ must be corrected by subtracting the delay d in order to adequately relate it to the stimulus position.^5^ The corrected phase φ^+^ = ϕ^+^ - d then indicates the true retinotopic location of the stimulus that evoked the response. The procedure for determining this delay is described in Step 19.
  e. Generate the resulting phase map ϕ^+^ (Figure 3C) showing the retinotopic position modulated by hemodynamic delay, and the magnitude map (Figure 3D) showing signal strength at each pixel. Troubleshooting problem 4.

19. Conjugation of Forward and Reverse Responses to remove the hemodynamic delay.
  a. As φ^+^ and φ^-^ represent the true retinotopic map for forward and reverse stimulations, they are symmetric and φ^+^= - φ^-^.
    Given the equations φ^+^ = φ^+^ + d (Step 17d) and ϕ^-^ = φ^-^ + d (Step 18b), it follows that.
    ϕ^+^ + ϕ^-^ = φ^+^ + d + φ^-^ + d = 2d.
    Therefore, the hemodynamic delay d can be estimated as.
    d = (ϕ^+^ + ϕ^-^)/2.
    This yields a delay map that reflects homogeneous hemodynamic delay across the SC (Figure 3J).
    ***Note:*** The delay term d is represented in phase units within the range [0, 2π) (Figure 3J). When expressed in time units, phase values are converted according to (d / 2π) × T, where T is the stimulus period. This conversion yields the hemodynamic delay in seconds.
  b. Similarly, the absolute retinotopic phase map (Figure 3K) and its associated magnitude map (Figure 3L) are computed from the difference of phase maps (ϕ^+^ − ϕ^-^)/2, which is free of the hemodynamic delay.
    ***Note:*** The same protocol applies to azimuth mapping, using vertical bar stimuli (Figure S2).

### Reconstruction of the orientation map

**Timing: 10 min**

**Figure 4:**
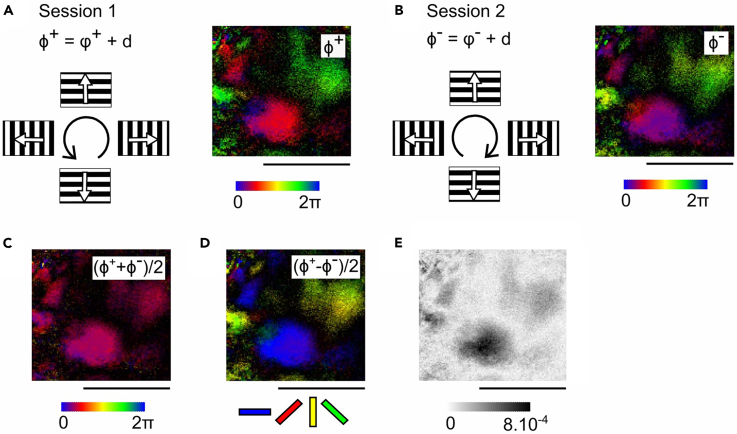
Fourier-based orientation mapping in the superior colliculus (A) Left panel: Schematic representation of the forward stimulus. Oriented, drifting gratings are rotated periodically counterclockwise. The resulting measured phase Φ^+^ at peak amplitude reflects the sum of the true neuronal response phase ϕ^+^ and the delay d, such that Φ^+^ = ϕ^+^ + d. Right panel: The reconstructed phase map Φ^+^ computed from the Fourier transform of the pixelwise signal during forward stimulation. (B) Same as (A) for the reverse stimulus in which the oriented, drifting gratings are rotated periodically clockwise, yielding the measured phase map Φ^-^. (C) Delay map computed as (Φ^+^ + Φ^-^)/2, which reflects the hemodynamic delay across the visual field. (D) Orientation phase map computed as (Φ^+^ − Φ^-^)/2, which is free of the hemodynamic delay. (E) Magnitude map showing the strength of orientation-evoked response. Scalebar: 1 mm.

This section describes how to generate orientation maps from periodic stimulation using Fourier analysis, following a method analogous to that used for retinotopic mapping. Here, the periodic stimulation consists of gratings drifting in one direction that are rotated continuously for 30 s, either anticlockwise (Figure 4A) or clockwise (Figure 4B). The drift rate of the gratings is set to 1.5 Hz and the spatial frequency to 0.015 cycles per degree.

20. Analysis of orientation signals.
  a. For each pixel, extract the response at the frequency of orientation presentation using Fast Fourier Transform (FFT). The relevant frequency is twice the frequency of direction presentation, i.e., 1/15 Hz.
  b. Compute the respective phase maps ϕ^+^ and ϕ^-^, which reflect the orientation-selective responses along with the hemodynamic delay, as well as the corresponding magnitude maps, representing the strength of orientation-selective responses (Figures 4A and 4B).
  c. Combine the two maps by computing the half-sum (ϕ^+^ + ϕ^-^)/2, which isolates the hemodynamic delay (Figure 4C), and the half-difference (ϕ^+^ − ϕ^-^)/2, which yields the corrected orientation phase map (Figure 4D) and its associated magnitude map (Figure 4E).

***Note:*** To validate the Fourier-based orientation map, one can compare it with response maps obtained from classical episodic stimulation (i.e. discrete trials of single orientations). As shown in Figure S3, subtracting orthogonal orientation responses reveals domains that spatially correspond to those obtained from periodic mapping. Quantification of signal intensity within these domains confirms the consistency between the two methods, supporting the reliability of the periodic Fourier-based approach. Troubleshooting problem 4.

### Surgical procedure to expose the binocular primary visual cortex and preparation of the imaging setup

**Timing: 30 min**

**Figure 5:**
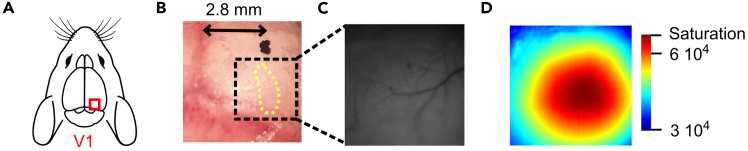
Surgical procedure and illumination for intrinsic optical imaging of the binocular visual cortex (A) Schematic representation of the binocular region of primary visual cortex (V1b) and its exposure for intrinsic optical imaging. (B) Illustration showing the estimated location of V1b (yellow dotted outline). A black dot is marked approximately 2.8 mm lateral to the midline to aid in precise camera positioning over the binocular zone. (C) Image of the V1b region acquired under green light illumination, revealing cortical vasculature and surface landmarks for targeting. (D) Same field of view as in (C), but imaged under red light illumination, optimized for intrinsic signal acquisition during functional mapping.

***Note:*** Hemodynamic signals can be recorded through the skull, a craniotomy is not necessary.

21. Make a skin incision and expose the scalp, as previously described (see Step 6 and Figure 2C).
22. Measure a distance of 2.8 mm lateral from the sagittal suture and anterior to the lambda, and mark the location on the skull (Figures 5A and 5B). This point serves as a reference for the position of the binocular primary visual cortex, without obstructing the region.

23. Apply agarose.
  a. Put a coverslip directly on the skull to prevent drying during the acquisition.
  b. Remove the eyelid of both eyes as described in Steps 12 and 13.

***Alternatives****:* Intrinsic signals are recorded through the intact skull in this protocol. However, skull thinning is also possible and may improve signal quality. In this case, thinning should be done cautiously to avoid damaging underlying vasculature or overheating the bone.

24. Prepare the imaging setup as described in Steps 14–16.
  a. Use the mark made in Step 22 to position the camera above V1 (Figure 5C) using the green filter.
  b. For the recording session, using the red filter, adjust the illumination to reach ∼5–10% below the saturation level (Figure 5D).

### Reconstruction of the ocular dominance map in the binocular primary visual cortex

**Timing: 40 min**

This section describes how to compute ocular dominance (OD) map from intrinsic optical signal data recorded in the binocular region of V1 using independent, periodic stimulation of the contralateral and ipsilateral eyes.

25. Visual stimulation in the binocular field of the contralateral (Figures 6A–6C) and the ipsilateral (Figures 6D–6F) eyes.
  a. Place the stimulus monitor 20 cm in front of the mouse, with the center of the screen aligned to the vertical meridian of the head.
    **CRITICAL:** Ensure that both eyes are horizontally aligned in the stereotaxic frame. For that, check the head position from above to ensure it is level and the ear bars support both sides evenly. Misalignment will lead to different portions of the visual cortex being stimulated during contralateral and ipsilateral eye presentations, preventing accurate computation of ocular dominance maps. Troubleshooting problem 5.
  b. Occlude the non-stimulated eye using a black shield (see Step 15) to ensure monocular viewing.
  c. Stimulate the animal with a white bar drifting (2° width) along the vertical axis of the screen. The bar covers the binocular portion of the visual field (i.e., from −5° in the ipsilateral field to +15° in the contralateral field). Each cycle lasts 8 seconds and is presented periodically for a total of 30 repetitions.
  d. Stimulate each eye in alternating blocks. The stimulation is organized into 10 consecutive blocks, with the contralateral eye stimulated in odd-numbered blocks (#1, #3, #5, #7, #9; see Figure 6B), and the ipsilateral eye in even-numbered blocks (#2, #4, #6, #8, #10; see Figure 6E). This results in 5 repetitions per eye, allowing 5 estimates of the ocular dominance map in the same animal.

**Figure 6:**
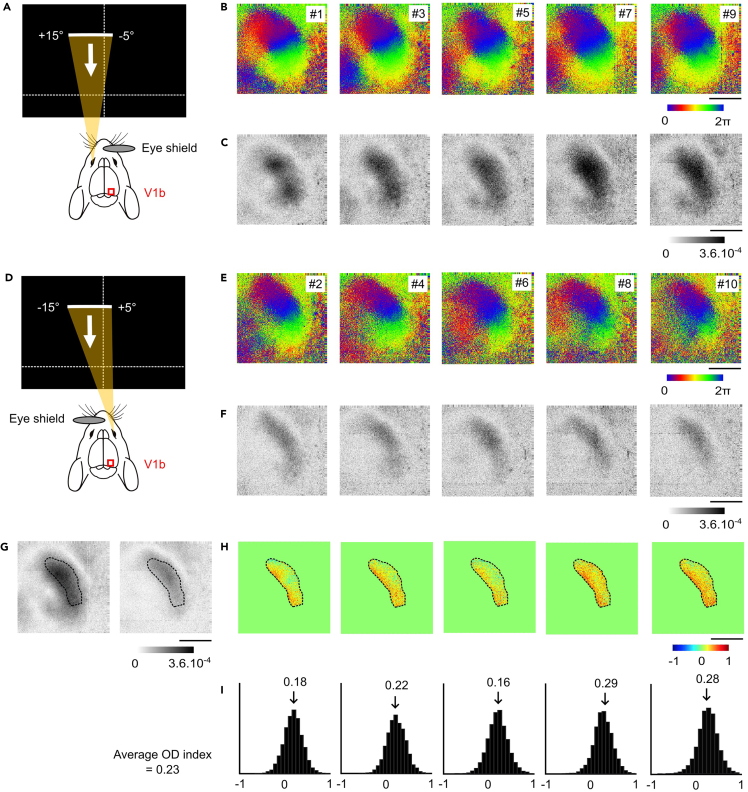
Application of intrinsic optical imaging to map ocular dominance in the binocular visual cortex (A) Schematic representation of the stimulated region of visual space spanning from +15° in the contralateral hemifield to −5° in the ipsilateral field. During this condition, the right eye is occluded using an eye shield and visual stimulation is applied to the left (contralateral) eye. (B–C) Retinotopic maps of (B) phase and (C) intensity obtained from five independent imaging sessions using contralateral eye stimulation. (D–F) Same as (A–C), but using ipsilateral eye stimulation while the left eye is occluded. (G) Average magnitude maps across sessions for contralateral stimulation (left panel) and ipsilateral stimulation (right panel). A region of interest (ROI) corresponding to the binocular zone is outlined with a black dotted line based on the overlap between both response fields. (H) Ocular dominance maps computed across five repetitions, with red areas showing contralateral preference and blue areas showing ipsilateral preference. (I) Histograms showing the distribution of ocular dominance (OD) scores across all pixels within the binocular zone for each of the five OD maps shown in (H). The vertical arrows indicate the mean OD score (OD index) for each session. Scalebar: 1 mm.
26. Reconstruction of the Ocular dominance maps.
  a. Compute the phase and magnitude retinotopic maps separately for each individual stimulation block using the same method described in Step 17. This results in 10 individual retinotopic maps (5 for the contralateral eye, Figures 6B and 6C and 5 for the ipsilateral eye; Figures 6E and 6F).
  b. For each eye, average magnitude maps across multiple imaging sessions (Figure 6G) and define the binocular zone in which both eyes elicit robust responses (Figure 6G, dotted outline).
    ***Alternatives****:* Since the stimulus was specifically restricted to the binocular field, the average magnitude map for the ipsilateral eye is often sufficient to define the binocular zone. However, keep in mind that this assumption is based on the typical organization observed in adult wild-type mice, and the extent of the binocular field may vary in transgenic lines or during postnatal development.
    ***Note:*** To improve visualization of the cortical region activated by each eye, the averaged magnitude maps can be smoothed using a Gaussian low-pass filter (e.g., 3 pixels standard deviation). A threshold can also be applied to eliminate background noise and isolate robust response regions. Threshold values in the literature typically range between ∼30–40% of the maximum response^14^ to the 50% threshold^8^ used here. Troubleshooting problem 4 and problem 5.
  c. Calculate the ocular dominance (OD) maps. For each imaging session pair (contralateral and ipsilateral stimulation), compute an OD score for each pixel within the binocular zone using: OD score = (C − I)/(C + I) where C and I are the response magnitudes to contralateral and ipsilateral eye stimulation, respectively (Figure 6H).
  d. For each OD map, compute the OD index as the mean OD score across all pixels in the binocular zone (Arrows in Figure 6I).
  e. Average the OD indices across all five repetitions to obtain a robust estimate.

## Expected outcomes

This protocol enables the generation of functional retinotopic and orientation maps in the superior colliculus (SC) and visual cortex (V1), providing a powerful tool to study how visual space is represented and organized across large portions of individual brain areas. These maps allow investigation of how topographic organization supports sensory processing, and how multiple functional features, such as position, orientation, and eye dominance, are spatially aligned.^5^^,^^13^^,^^24^

This protocol has proven effective for examining the molecular and activity-dependent mechanisms that shape visual maps. In developmental studies, it has, for instance, been applied to investigate ephrin signaling and topographic precision in both the SC and V1^16,17^. Moreover, this method not only captures the topology of functional maps, but also reveals differences in signal amplitude that can reflect physiological or cellular modulation. For example, impairing astroglial coupling reduces the magnitude of visual responses in the SC while sparing V1, illustrating the region-specific contribution of astrocytes to sensory processing.^1^

In the binocular zone of V1, this protocol enables straightforward assessment of ocular dominance plasticity by independently stimulating each eye and computing an ocular dominance index (ODI). A brief period of monocular deprivation is indeed sufficient to reveal an imbalance in the cortical representation of each eye, provided that the brain is still capable of plastic change. This makes the method ideally suited to investigate the mechanisms that govern the critical period, as well as experimental strategies to reopen plasticity in the adult brain. It has been used in this context to investigate how specific cell types, such as inhibitory neurons,^25^ astrocytes,^8^ or oligodendrocytes,^23^ and physiological or environmental conditions, such as dark exposure^26^ or enriched environments,^11^ shape cortical plasticity.

## Quantification and statistical analysis

The analysis pipeline for retinotopic mapping is illustrated using the dataset corresponding to the example shown in Figures 3A–3C. It corresponds to Step 17 of the section “Analysis of the retinotopic map from forward and reverse stimulations.” This dataset (Downsized for GitHub), along with a MATLAB script, is provided https://github.com/tchetch12/IOI_Retinotopy_Analysis.^27^ The provided script performs the following key steps.

### Preprocessing and pre-filtering

The raw optical signals (Raw_data.mat) are organized into a 2D matrix (time x space). The Generalized Indicator Function (GIF) method is then applied to discard non-stimulus related signals from the data.

### Fourier analysis

A pixel-wise Fourier transform is computed to extract the complex response at the stimulus frequency. The amplitude reflects the response strength, while the phase encodes the peak of the hemodynamic response.

## Limitations

Intrinsic optical imaging captures variations in cortical reflectance that reflect changes in blood volume and oxygenation rather than direct neuronal activity. While this allows for large-scale functional mapping, the signal is shaped by multiple non-neuronal contributors such as astrocyte dynamics, vascular tone, and metabolic coupling.^3^^,^^6^^,^^28^ As a result, changes in the intrinsic signal may not necessarily reflect changes in spiking activity, and interpretations must be made with caution, particularly in models involving glial or vascular alterations.

The technique is inherently limited to surface-accessible brain structures. In cortical areas such as V1, imaging is straightforward, but in subcortical targets like the superior colliculus, surgical aspiration of overlying cortex is required. Moreover, due to light scattering and depth integration, the recorded signal arises from an extended cortical volume,^2^^,^^29^ making it difficult to attribute responses to specific laminar compartments.

Although the method offers high spatial resolution across large areas, it does not provide single-cell resolution, and cannot resolve microcircuit-level activity. It is therefore best combined with complementary techniques such as electrophysiology or calcium imaging when cellular specificity is needed.

Finally, this approach relies on periodic and stable stimulation paradigms. It is well suited to mapping structured representations such as retinotopy and orientation preference, but is not optimal for studying responses to complex, dynamic, or behaviorally relevant stimuli, which require finer temporal resolution and more flexible stimulus control.

## Troubleshooting

Here, we summarize the problems which can occur during the different steps of the protocol and provide potential solutions.

### Problem 1

Corneal opacity compromising visual stimulation (step 5).

Prolonged surgical duration, especially during superior colliculus (SC) preparation, may lead to corneal dehydration. If the cornea is not frequently rehydrated with saline, it may become opaque and lose transparency, compromising visual stimulation fidelity.

### Potential solution

During all surgical steps, try to apply a drop of 0.9% NaCl solution regularly onto both eyes to keep the cornea moist. If whitening or opacity begins to appear, apply a large drop of saline on the affected eye and wait for 5–10 minutes. In some cases, rehydration may restore clarity. Alternatively, a transparent eye ointment or mineral oil can be used to maintain corneal hydration for longer periods.

If the cornea remains opaque, the damage may be irreversible. For V1 imaging, where only one eye is stimulated, consider switching to the contralateral hemisphere corresponding to the healthy eye. For SC imaging, which requires prolonged preparation, it may be preferable to terminate the experiment to avoid restarting the entire procedure. Likewise, for OD mapping in V1, which requires reliable stimulation of the two eyes, ending the session is recommended.

***Note:*** In rare cases, a white or opaque cornea may indicate pupil dilation due to respiratory arrest. Always verify that the animal is still breathing normally before concluding corneal damage.

### Problem 2

Persistent bleeding during bone flap removal and SC exposure (step: 9).

During removal of the skull overlying the superior colliculus (SC), bleeding can occur from dural sinuses, particularly at the intersection between the transverse and sagittal sinuses. This may obstruct the surgical field and hinder access to the SC.

### Potential solution

First, prevent this issue by ensuring that the bone flap is evenly thinned before lifting and by avoiding any tearing motion that could disrupt underlying vessels.

If it happens, apply a small piece of Spongel soaked in cold Ringer’s solution directly onto the bleeding site. Press gently with a sterile tissue (e.g., Kimtech) to stabilize it. Apply a continuous flow of ice-cold Ringer’s solution or PBS as cold exposure lead to vasoconstriction.

Once the bleeding has stopped, if the Spongel obstructs the region that must be cleared for imaging or aspiration, do not attempt to remove it directly, as bleeding may resume. Instead, use fine tweezers to gradually push it aside while maintaining pressure on the vessel. If needed, cut the Spongel into small pieces with fine scissors to expose the cortex gradually without provoking re-bleeding.

In extreme cases where a transverse sinus is injured and bleeding cannot be controlled by standard means, ligation and transection of the vessel can be performed as described in previous protocols.^30^ This should only be done by experienced surgeons, as it carries significant risk.

### Problem 3

SC is not clearly visible after cortical aspiration (step: 11).

Following cortical aspiration, the superior colliculus (SC) may not be clearly visible. This is often due to incomplete removal of the overlying visual cortex, especially in its anterior part.

### Potential solution

Continue aspiration carefully until the full anterior–posterior extent of the cortex overlying the SC has been removed. The surface of the SC is typically identifiable by the presence of fine, parallel blood vessels, and the specific inclination of this structure; anterior part is deeper than the posterior segment.

Perform the procedure progressively and use low vacuum pressure to avoid damaging the SC surface. Start at the lowest effective vacuum setting and adjust gradually while observing the tissue, increasing pressure only as needed to remove cortical tissue carefully without deforming or damaging the SC.

Adapt the angle and depth of aspiration to match the natural slope of the SC.

In case of uncertainty, consider gently rinsing with cold Ringer’s solution to remove remaining tissue fragments and improve surface contrast.

### Problem 4

Weak or absent intrinsic signal (steps: 17, 20, 26).

A low signal-to-noise ratio can prevent reliable extraction of phase and magnitude components and thus impair retinotopic or orientation map quality. This is a common issue, especially during early attempts or when animal physiology or optics are suboptimal.

### Potential solution

Verify the imaging setup: ensure that the red filter is correctly installed and check that the light intensity has not increased above the camera’s saturation level. Also confirm that the region of interest is correctly defined and centered over the target structure.

Check the animal’s physiological state: a stable plane of anesthesia and core body temperature at ∼37°C are essential. Monitor respiratory rate and adjust anesthetic levels as needed.

Evaluate the optical interface: make sure that the agarose layer is homogenous and that the coverslip sits flat on the brain surface without bubbles.

Consider the skull condition: in V1 imaging, the signal strength may vary across preparations depending on skull clarity. In such cases, thinning the skull slightly may improve the signal.

Reassess stimulation parameters: confirm that visual stimuli are correctly timed, synchronized with the acquisition system, and sufficiently salient (contrast, luminance, size).

### Problem 5

Misaligned or inconsistent ocular dominance (OD) maps between eyes (steps: 25, 26).

OD maps may appear spatially shifted, distorted, or non-overlapping between contralateral and ipsilateral eye stimulation. This inconsistency can prevent accurate computation of OD values.

### Potential solution

Check the alignment of the eyes in the stereotaxic frame. Both eyes must be horizontally aligned to ensure that visual stimuli activate the same region of the visual cortex during monocular presentations. Even a slight tilt can result in shifted retinotopic maps. In practice, alignment is typically verified visually from above, ensuring that the head is level and that the ear bars do not lift one side of the head.

Verify the position and stability of the stimulus monitor: it should remain centered on the vertical meridian across all sessions. Use fixed distance markings to reproduce identical screen positioning across animals and conditions.

Ensure the occlusion of the non-stimulated eye is complete to avoid cross-contamination of responses. Any residual light input from the occluded eye may reduce the signal specificity and blur OD boundaries.

Normalize and threshold the magnitude maps before OD calculation to focus analysis on the overlapping binocular zone. Minor differences in signal strength between the two eyes can lead to apparent OD shifts if not correctly masked.

## Resource availability

### Lead contact

Further information and requests for resources and reagents should be directed to and will be fulfilled by the lead contact, Ribot Jérôme (jerome.ribot@college-de-france.fr).

### Technical contact

Technical questions on executing this protocol should be directed to and will be answered by the technical contact, Ribot Jérôme (jerome.ribot@college-de-france.fr).

### Materials availability

This study did not generate new reagents.

### Data and code availability

Example dataset (retinotopic maps from Figure 3) and all custom analysis code supporting this study have been deposited in GitHub at https://github.com/tchetch12/IOI_Retinotopy_Analysis.

## Acknowledgments

This work was supported by grants from 10.13039/501100004431Fondation de France to F.B.-P., 10.13039/501100002915Fondation pour la Recherche Médicale (FRM) to J.V. and N.R., the 10.13039/501100000781European Research Council (Consolidator grant no. 683154), the European Union’s Horizon 2020 Research and Innovation Program (Marie Sklodowska-Curie Innovative Training Networks, grant no. 722053, EU-GliaPhD), the 10.13039/501100001665ANR Chaire D’Excellence (ANR-24-CHBS-0007), and the Major Research Program of PSL Research University “PSL-Neuro,” launched by PSL Research University and implemented by 10.13039/501100001665ANR (ANR-10-IDEX-0001), to N.R.

## Author contributions

Conceptualization and optimization of the protocol, J.V., F.B.-P., and J.R.; investigation, F.B.-P., A.M., J.V., and J.R.; software, J.R.; validation, F.B.-P., A.M., J.V., N.R., and J.R.; writing – original draft, F.B.-P. and J.R.; writing – review and editing, all authors; visualization, F.B.-P. and J.R.; supervision, J.R., A.R., and N.R.

## Declaration of interests

The authors declare no competing interests.
