# Supplementary material for: Protocol for recording visual maps in the mouse superior colliculus and visual cortex with intrinsic optical imaging
Source: STAR Protoc. 2026 Mar 12;7(1):104430. doi: 10.1016/j.xpro.2026.104430 (PMC12990355; doi:10.1016/j.xpro.2026.104430)
Supplement: Document S1. Figures S1–S3 [file mmc1.pdf]

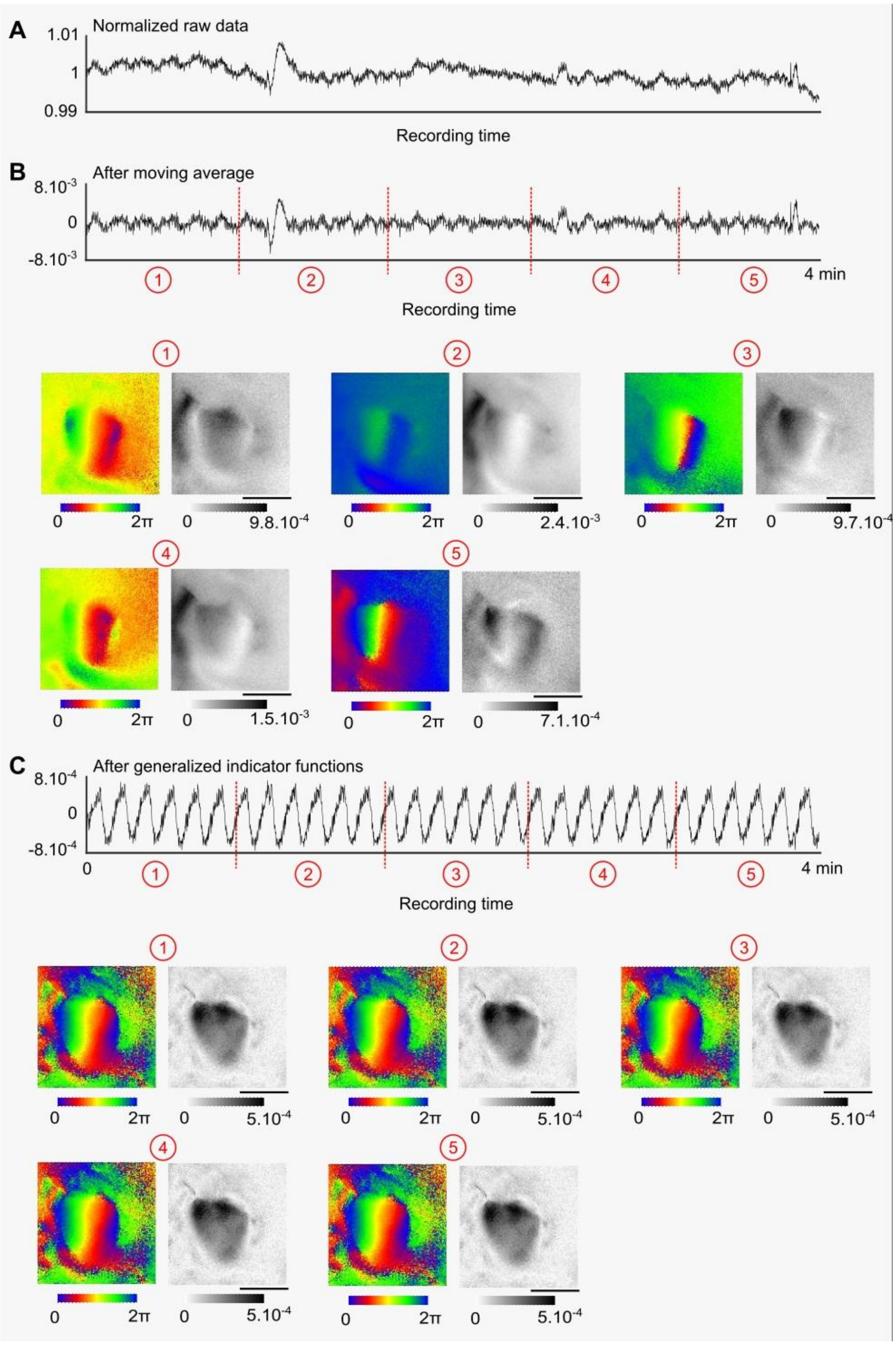

Figure S1. Temporal Pre-filtering Improves Retinotopic Map Stability in the Superior Colliculus, related to Step 17.

(A) Temporal signal from a single pixel during forward stimulation, shown after normalization by its temporal mean. This is the same raw trace as in **Figure 3E (top panel)**.

(B) Same signal as in (A), after **moving average filtering** (as in Figure 3E, middle panel). The signal was segmented into five equal-duration epochs. For each epoch, a **phase map** and a **magnitude map** were computed using the standard Fourier approach. The resulting maps show substantial variability across segments, reflecting instability due to remaining noise in the signal.

(C) Same analysis as in (B) but applied to the signal preprocessed using the **generalized indicator function method** (as in Figure 3E, bottom panel). Phase and magnitude maps reconstructed from each segment show high consistency and stability across time, highlighting the improved robustness of this pre-filtering method.

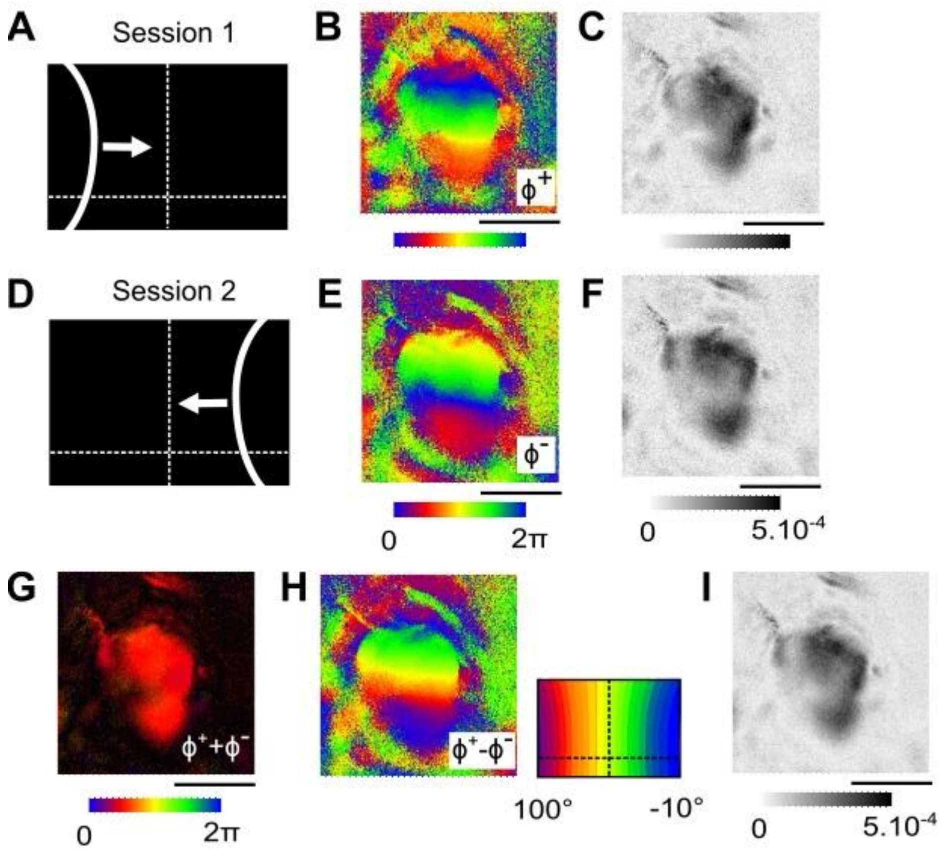

Figure S2. Fourier-Based Retinotopic Mapping of Azimuth in the Superior Colliculus, related to Step 19.

(A) Example of visual stimulation for the **forward condition**, in which the stimulus moves **from left to right**, systematically sweeping azimuthal positions across the visual field.

(B) Resulting **phase map (Φ^+)** computed from the pixelwise Fourier transform of the signal during forward stimulation, showing azimuthal organization combined with hemodynamic delay.

(C) Corresponding **magnitude map**, indicating the signal strength at the stimulation frequency for each pixel.

(D–F) Same as (A–C), but for the **reverse condition**, in which the stimulus moves **from right to left**. This yields the measured phase map Φ^- and corresponding magnitude.

(G) Sum of the two-phase maps ($\Phi^+ + \Phi^-$), representing **twice the hemodynamic delay** across the imaged field.

(H) Difference of phase maps ($\Phi^+ - \Phi^-$), revealing the **true retinotopic phase map** for azimuth after delay correction.

(I) Magnitude map associated with the corrected phase in (H).

Scalebar: 1 mm

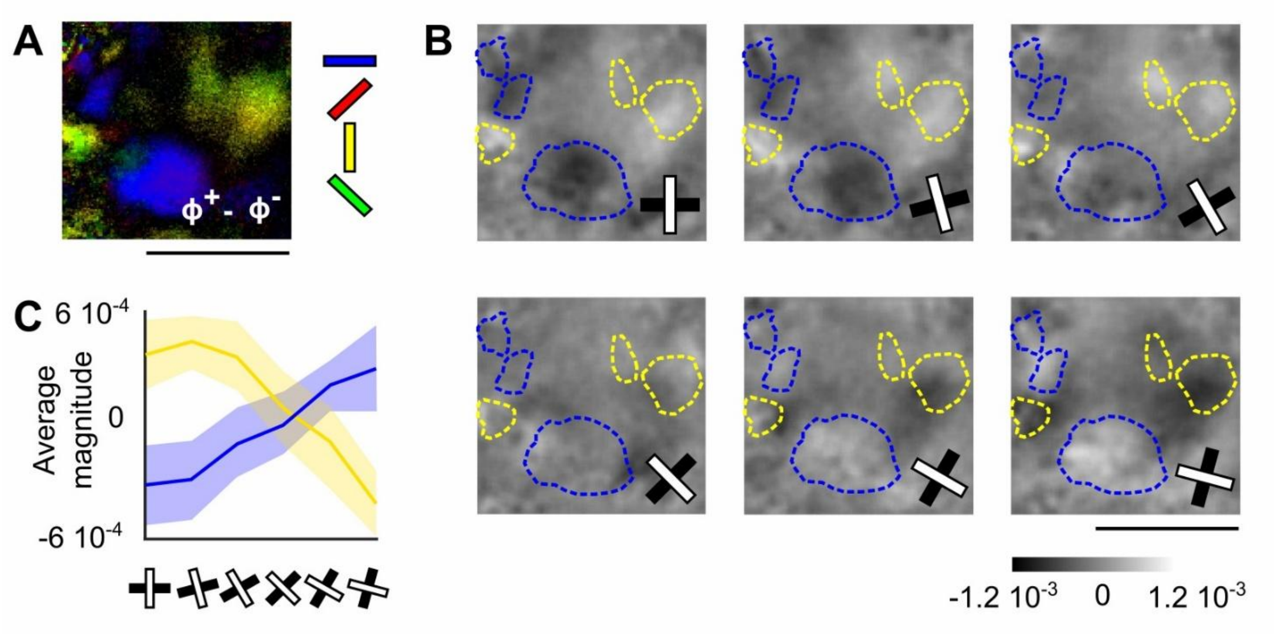

Figure S3. Comparison of Fourier-based Orientation Mapping in the Superior Colliculus with Episodic Stimulation, related to Step 20.

(A) Rectified orientation map obtained from periodic stimulation and Fourier analysis, as shown in Figure 4. Vertical and Horizontal orientation domains were extracted from this map and used as reference regions in (B–C).

(B) Comparison with responses to episodic stimulation of orientated gratings. Each map represents differential response to two orthogonal orientations. White and black areas indicate regions preferring one of the two orthogonal orientations. Dashed outlines represent orientation domains extracted from (A).

(C) Quantification of signal selectivity in functional domains. The mean signal (± standard deviation) of responses in (B) is computed within horizontal (blue) and vertical (yellow) orientation domains extracted from (A). Yellow domains show stronger responses to vertical gratings, and blue domains respond preferentially to horizontal gratings presentation, confirming the consistency of the Fourier-based orientation map with the classical episodic stimulation method.

Scalebar: 1 mm
